# Quantitative assessment of the lumbar intervertebral disc via T2 shows excellent long-term reliability

**DOI:** 10.1371/journal.pone.0249855

**Published:** 2021-04-14

**Authors:** Daniel L. Belavy, Patrick J. Owen, Gabriele Armbrecht, Martin Bansmann, Jochen Zange, Yuan Ling, Regina Pohle-Fröhlich, Dieter Felsenberg

**Affiliations:** 1 Deakin University, Spine Research Group, Institute for Physical Activity and Nutrition, School of Exercise and Nutrition Sciences, Geelong, Victoria, Australia; 2 Charité–Universitätsmedizin Berlin, Corporate Member of Freie Universität Berlin, Humboldt-Universität zu Berlin, and Berlin Institute of Health, Institute of Radiology, Berlin, Germany; 3 Division of Physiotherapy, Department of Applied Health Sciences, Hochschule für Gesundheit (University of Applied Sciences), Bochum, Germany; 4 Krankenhaus Porz am Rhein, Institute for Diagnostic and Interventional Radiology, Krankenhaus Porz am Rhein, Cologne, Germany; 5 German Aerospace Centre, DLR e.V., Linder Höhe, Cologne, Germany; 6 Imaging at Olympic Park, Melbourne, Victoria, Australia; 7 Hochschule Niederrhein, Graphische Datenverarbeitung und Bildverarbeitung, Krefeld, Germany; University of Toronto, CANADA

## Abstract

Methodologies for the quantitative assessment of the spine tissues, in particular the intervertebral disc (IVD), have not been well established in terms of long-term reliability. This is required for designing prospective studies. ^1^H water T_2_ in the IVD (“T_2_”) has attained wider use in assessment of the lumbar intervertebral discs via magnetic resonance imaging. The reliability of IVD T_2_ measurements are yet to be established. IVD T_2_ was assessed nine times at regular intervals over 368 days on six anatomical slices centred at the lumbar spine using a spin-echo multi-echo sequence in 12 men. To assess repeatability, intra-class correlation co-efficients (ICCs), standard error of the measurement, minimal detectable difference and co-efficients of variation (CVs) were calculated along with their 95% confidence intervals. Bland-Altman analysis was also performed. ICCs were above 0.93, with the exception of nuclear T_2_ at L5/S1, where the ICC was 0.88. CVs of the central-slice nucleus sub-region ranged from 4.3% (average of all levels) to 10.1% for L5/S1 and between 2.2% to 3.2% for whole IVD T_2_ (1.8% for the average of all levels). Averaging between vertebral levels improved reliability. Reliability of measurements was least at L5/S1. ICCs of degenerated IVDs were lower. Test-retest reliability was excellent for whole IVD and good to excellent for IVD subregions. The findings help to establish the long-term repeatability of lumbar IVD T_2_ for the implementation of prospective studies and determination of significant changes within individuals.

## Introduction

With a long-term view to automated radiographic analysis of spinal structures on magnetic resonance imaging (MRI), establishing methodologies for quantification of spinal structures is a key step. For example, intervertebral disc (IVD) degeneration is currently assessed by semi-quantitative grading scales, such as the Pfirrmann grading scheme [1]. These scales are insensitive to subtle changes in the IVD [2] and in the long-term, similar to current assessment of bone mineral density in the spine, a population based reference to which to compare the IVDs of an individual to determine the degree of IVD degeneration would be preferable. The expertise in the field for quantitative MRI of the IVD is improving: approaches for automated identification and segmentation of the lumbar IVD [3] and methods to quantify the IVD on MRI [4] are being established. The assessment of IVD T_2_ relaxation time is, arguably, currently the most widely studied approach, with works on comparison of T_2_ to the composition and structure [4–7] of the IVD and to IVD degeneration [8–11]. In an IVD the T_2_ value of water ^1^H is predominantly determined by the mobility of water, which depends on the rapid exchange between free mobile water and the immobile water of the hydration shell of macromolecules like glycosaminoglycan and collagen. In so far T_2_ is a qualitative marker for the relative water and protein content in the IVD. If, for example the volume of an IVD increases with a parallel increase in T_2_, this volume increase would predominantly result from water uptake. A volume increase at constant T_2_ would indicate a proportional water uptake and protein synthesis.

To accept a methodology for wider use, it is important to understand its reliability; that is, how much measurement error exists when scanning the same person one time-point to the next. This then drives the responses to key questions of, for example, sample size estimation in study design [12] or whether the change seen in an individual over time is ‘more than measurement error’ (i.e. minimum detectable change). The reliability of IVD T_2_ measurements has received limited attention. In our search of the literature, we were able to identify only one study [13] that examined the reliability of T_2_ measurements of the IVD in (living) human participants where the sequence included four echos in 25 and 100ms; thus, leading to a less precise estimation of T_2_ than currently possible. This study examined reliability by performing scanning 15 minutes after the first scan in 10 participants. These authors found that the measurement variability was higher at the lower lumbar spine in comparison to the upper lumbar spine. Given that the adaptation of the IVD is a slow process [14, 15], and long-term reliability is typically worse than the short-term reliability, it is important to know what the long-term reliability of IVD T_2_ measurements are. In this work, we examine the long-term reliability of IVD T2 over 1.5 years.

## Materials and methods

### Study design and participants

Twelve males aged 20–44 years participated in the study which was conducted as part of a wider project examining the spine in repeated bouts of bed-rest [16]. Participant mean (SD) age, height and weight were 34.3(8.3)yr, 176.2(5.9)cm and 69.8(8.0)kg respectively. Nine scanning sessions were performed over 1yr: baseline, 34d, 60d, 154d, 188d, 214d, 308d, 342d and 368d post-baseline. For assessing reliability using nine repeated measurements, it is recommended that at least four participants are measured [17]; thus, our design ensured an adequate assessment of measurement reliability. The participants were tested at a university hospital. Exclusion criteria included a history of chronic low back pain, current episode of low back pain or any known genetic muscle and bone diseases. None of the participants had a history of spinal surgery. This study was carried out in accordance with the Declaration of Helsinki guidelines for research on human participants (1989). Ethical oversight and approval was provided by the *Comite de Protection des Personnes Sud-Ouest et Outre-Mer I* (CPP SOOM I; Toulouse, France. Id number B120707-48). All participants gave their informed written consent prior to involvement in the study.

### Magnetic resonance imaging-protocol

To minimise the impact of normal diurnal variation [18] on the study, all scanning was performed after 20:00 hours to control for the influence of body-fluid shifts [19] participants were required to remain in lying for 2hr prior to the start of the scanning procedure. Participants were transferred in lying to the scanning table and positioned in supine lying in the scanner with a standard cushion behind their knees and a standard pillow behind their head. A spin-echo multi-echo sequences on a 1.5T Siemens Avanto scanner (Erlangen, Germany) was used with the body coil to collect images at 16 echo times (10.9, 21.8, 32.7, 43.6, 54.5, 65.4, 76.3, 87.2, 98.1, 109, 119.9, 130.8, 141.7, 152.6, 163.5 and 174.4ms) from six sagittal anatomical slices each (thickness: 3mm; interslice distance: 3mm; repetition time: 2500ms; field of view: 300x300mm; image resolution: 0.781mm/pixel) centred at the lumbar vertebrae. To enable Pfirrmann grading of IVD degeneration at the first scanning session, a T2-weighted sequence (thickness: 3mm; interslice distance: 0.3mm; repetition time: 8740ms; echo time: 104mm; field of view: 380x380mm; image resolution: 1.188mm/pixel) encompassing the entire lumbar spine with 29 sagittal anatomical slices was also collected. Images were exported for further offline processing. Pfirrmann grading was performed on IVDs from baseline scans by a qualified radiologist. To ensure blinding of the examiner to prior measurements, each testing session was assigned a random number (obtained from www.random.org).

### Segmentation of IVDs and the semi-automated finding of five IVD subregions

**Fig 1:**
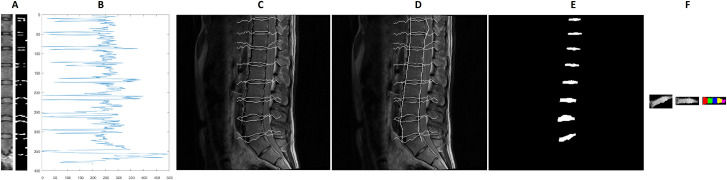
Semi-automated segmentation of the intervertebral discs. A: Sample subset of the spine and the corresponding detected contours (right), B: Grey values along the detected approximate centre of the spine, C: Detected contour following the vertebral end-plate, D: Including estimation of the anterior and posterior margins of the lumbar spine, E: Segmented intervertebral discs, F (left to right): Segmented intervertebral disc in original alignment, rotated towards the horizontal, subdivision into five sub-regions.

MATLAB (version R2018b; MathWorks; Massachusetts, USA) was used for all semi-automated image processing. For all anatomical slices, the images were automatically partitioned to include only the vertebral bodies and IVDs. The second echo from each anatomical slice was used for starting IVD segmentation as this showed the highest contrast. The third image (typically at the centre of the spine) was used first for localising the position of the IVDs. The image was first median-filtered (one pixel width, 15 rows; ‘median-image’). Then, for each pixel-column (i.e. at each x-axis coordinate, where the y-axis of the image is of the pixels in the cephalocaudal direction and the x-axis pixels in the anteroposterior direction) the difference in signal intensity units between the original image and the median-filtered image was calculated. All pixels 100 units or more than the median-filtered image were then labelled white and the remainder black (Fig 1A).

To localise the centre of the spine, the pixel-column which had the highest number of pixels with a signal intensity difference ≥100 units was taken. The signal intensity values of this pixel-column (Fig 1B) were then considered. The local minima with a signal intensity value of less than 150 units were taken as starting points. This identified, anatomically, the vertebral end-plate. Contour tracking (seeking a minimum of three neighbouring pixels with a signal intensity value <150 units) was performed anteriorly and posteriorly along the detected starting points (vertebral end-plates; Fig 1C).

At the next step, the anterior and posterior boundaries of the spine were delineated. First, for each pixel on each echo at a given anatomical slice the signal intensities are averaged (‘averaged-image’). Then the regions between each identified intervertebral disc (identified at previous step; ‘vertebral bodies’) were examined. The operator marked approximately eight support points per disc region in the middle layer. These were then connected optimally by means of graph search (Dijkstra algorithm). The deviation of the gradients from the gradient direction on the support points and the grey values were used as costs in the optimality function. The algorithm always provided the path with the lowest costs (i.e. the shortest possible connection along the object edges). Subsequently, the support points were propagated to the adjacent layers. For this purpose, a pixel with a similar gradient and grey value was searched in the environment of the current position in the neighbouring layer. Then the graph search algorithm is used again to find the optimal path between the new points. If necessary, the operator had the possibility to move the node manually to the best position. The whole process was repeated until all layers of a data set were segmented. The x-coordinate used as the starting point for contour tracking is again taken as the starting point. From this starting point in each pixel-row in each vertebral body, the algorithm searched left and right for the first instance of a signal intensity difference 80 units between the average-image and the median-image. This was then taken as the anterior and posterior border, respectively, of the spine (Fig 1D). Finally, the detected contours were filled and extracted, giving the locations of the IVDs (Fig 1E). The process was then repeated for the other anatomical slices.

To subdivide the respective disc into five equally sized segments anterior to posteriorly, the object orientation and the centroid were determined first by fitting an ellipse to the IVD, the angle between the major axis of the ellipse and the x-axis was noted and the IVD was then rotated to the x-axis. The width of the rotated object was measured and then divided into five segments of equidistant width (Fig 1F) and the mean grey values of the individual segments were determined. The coordinates of the region of interest for each IVD was applied to each echo and the signal intensity data were extracted for the whole IVD and subregions in each echo.

### Calculation of T2 values for each IVD subregion

**Fig 2:**
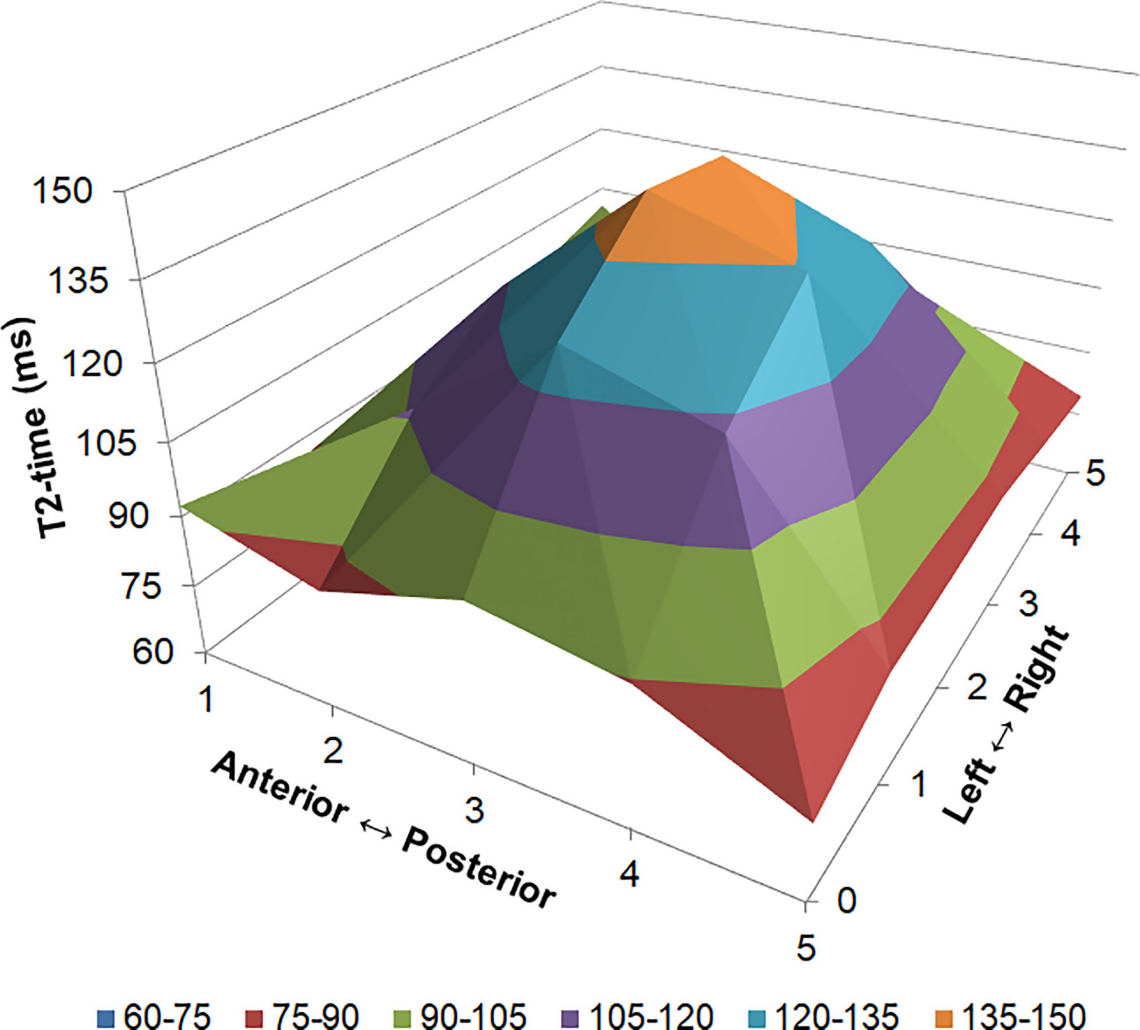
Three dimensional representation of T_2_ distribution in the lumbar intervertebral disc. Data have been averaged from all intervertebral levels, scanning dates and participants. Values are mean T_2_ (z-axis) in each of the five intervertebral disc subregions anterior to posterior (x-axis) and from on the six images from the left to right side of the spine (y-axis).

In each IVD and for each of the five predefined subregions the corresponding T_2_ was calculated using custom code written in the R statistical environment (version 3.4.2, www.r-project.org) from the decay of signal intensity measured at 16 different echo times (Fig 2). The primary analysis focussed on whole IVD T2 (T2 values of the whole IVD averaged across all anatomical slices) and T_2_ in the nucleus (T_2_ in the central subregion of the five IVD subregions in the third anatomical slice). Secondary analysis considered the reliability in the five IVD subregions from the anterior to posterior annulus, averaged across all images.

### Statistical analysis for the repeatability of T2 measurements

Following recommendations for studies of reliability [20], for each parameter the intra-class correlation coefficient (Type 1,1 according to Shrout and Fleiss [21] and Type 1 according to McGraw and Wong [22]), the standard error of the measurement (SEM = [SD of sample scores] * [square-root of {1-ICC}]) and the minimum detectable difference (MD = SEM*[square root of 2]*1.96) were calculated [23]. The minimum detectable difference is also termed the minimum detectable change (MDC) by other authors. Coefficients of variation (CVs) and their 95% confidence intervals (CIs) were also calculated as described elsewhere [17]. To test for agreement between pairs of testing days, Bland-Altman plots [24] were generated, the “limits of agreement” were calculated (as 1.96 times the standard deviation of the differences between testing days), and bias (e.g. overestimation of smaller values and underestimation of larger values) was assessed formally for the linear case via linear regression and the plots were also inspected visually for non-linear forms of bias. Earlier authors [25] have classified ICCs as poor, moderate, good or excellent when the ICC estimate was, respectively, less than 0.40, 0.40–0.59, 0.60–0.74 or greater than 0.75. To enable evaluation of the stability of T2 over differing spans of time, we also calculated the Pearson’s correlation coefficient of the T2 in the whole IVD and in the nucleus for all testing days. An alpha-level of 0.05 was assumed for statistical significance. All analyses were conducted in the “R” statistical environment (version 3.4.2, www.r-project.org) and the “irr” package was used for calculation of ICCs.

## Results

All twelve participants completed the first four testing time-points (baseline, 34d, 60d and 154d post-baseline), after this eleven were tested at 188d post-baseline, 10 at 214d and 308d post-baseline and eight at 342d and 368d post-baseline.

### Reliability

**Fig 3:**
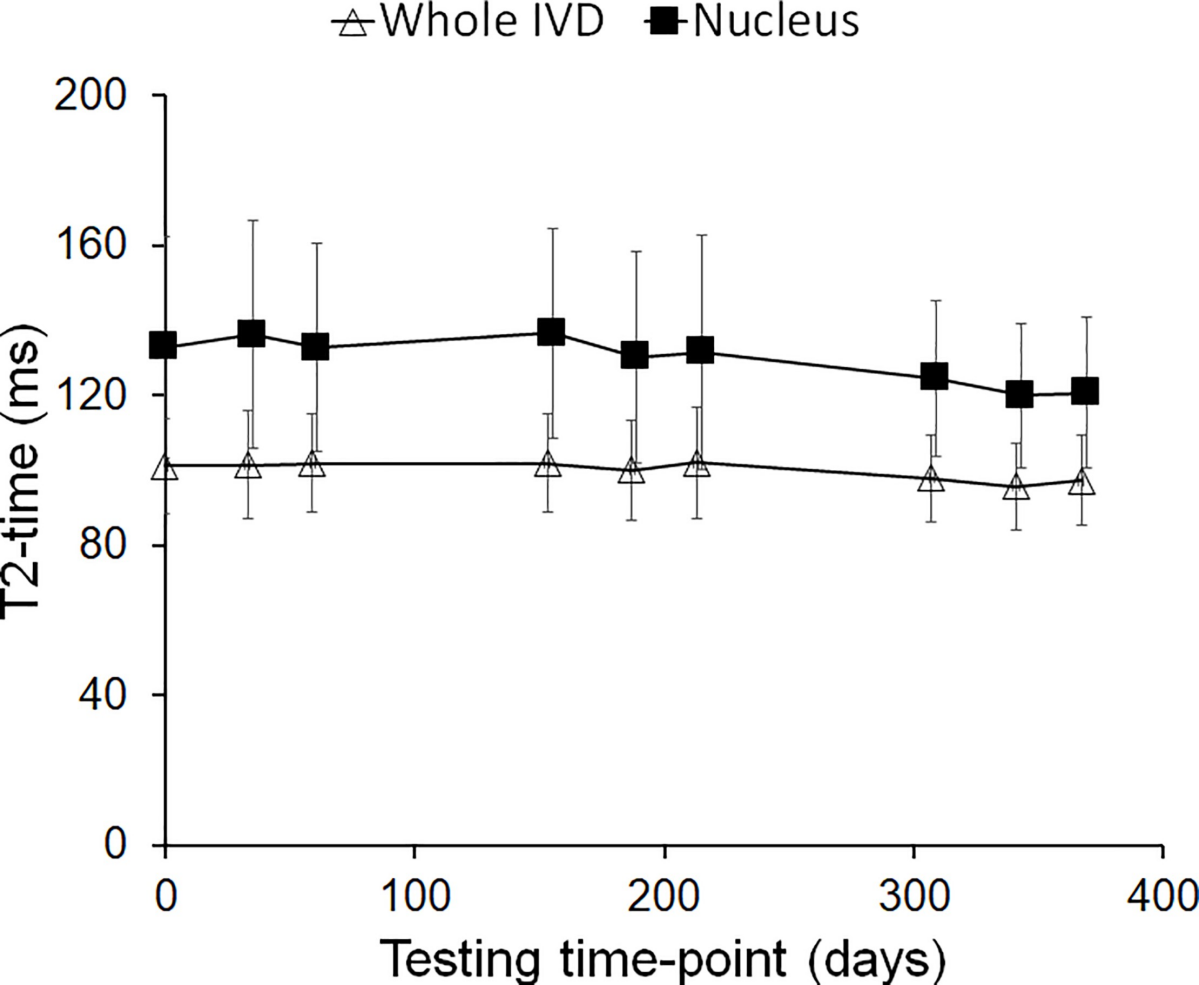
Whole and nuclear intervertebral disc (IVD) T2 across all scanning time-points. Values are mean (SD) of data averaged across all lumbar discs. Analysis of the limits of agreement (S1 Table) showed agreement between baseline and follow-up testing time-points. The subtle downward slope over time in both parameters was not significant (p>0.76) on further linear regression analysis.

For the whole IVDs and for the IVDs’ nucleus of the entire lumbar spine ICCs of T_2_ measured at different examination days (Fig 3) were above 0.93 (‘excellent’; Table 1) with the exception of nuclear T_2_ at L5/S1 where the ICC was 0.88 (‘excellent’). CVs were larger for the nucleus (central-slice only, subregion 3; 4.3% for the average of all levels to 10.1% for L5/S1) as compared to the T_2_ of the whole IVD (1.8% for the average of all levels and 2.2% [L2/3 and L3/4] to 3.2% [L5/S1]). Averaging between vertebral levels improved reliability, with significant improvements of CVs only (Table 1). Results from Bland-Altman analysis (S1 Table) showed agreement between all testing days for these parameters. In 7 of 96 cases (7.3%) there was some evidence of linear bias and none of these remained significant after controlling for false-positives by the false discovery rate method [26]. The reliability data for individual IVD subregions from the anterior annulus (subregion 1) to posterior annulus (subregion 5) are presented in Table 2.

**Table 1:** Whole (top) and nuclear (bottom) intervertebral disc T_2_ reliability.

Level	Global mean (SD)	ICC (95% CI)	SEM (95% CI)	MD (95% CI)	MD%	CV (95% CI)
*Whole IVD*						
AvLx	101.7(13.4)	0.98(0.95–1.00)	1.9(0.9–2.9)	5.1(2.5–8.0)	5.1	1.8(1.5–2.1)
L1/2	105.0(15.1)	0.97(0.94–0.99)	2.4(1.2–3.8)	6.8(3.3–10.5)	6.5	2.5(2.2–3.0)
L2/3	106.9(15.0)	0.98(0.95–0.99)	2.2(1.1–3.4)	6.1(3.0–9.5)	5.7	2.2(1.9–2.6)
L3/4	104.3(13.5)	0.97(0.93–0.99)	2.2(1.1–3.5)	6.2(3.0–9.6)	5.9	2.2(1.9–2.6)
L4/5	97.1(16.6)	0.97(0.93–0.99)	2.9(1.4–4.5)	8.1(4.0–12.5)	8.3	2.3(2.0–2.7)
L5/S1	95.1(18.6)	0.98(0.95–0.99)	2.7(1.3–4.3)	7.6(3.7–11.8)	8.0	3.2(2.8–3.8)
*Central slice*, *nuclear region*						
AvLx	134.4(28.9)	0.94(0.87–0.99)	6.9(3.4–10.5)	19.0(9.4–29.0)	14.2	4.3(3.7–5.0)
L1/2	127.6(33.3)	0.97(0.92–0.99)	5.9(2.9–9.1)	16.3(8.0–25.3)	12.8	4.9(4.2–5.8)
L2/3	141.7(29.9)	0.96(0.91–0.99)	6.0(2.9–9.2)	16.6(8.2–25.5)	11.7	4.8(4.2–5.7)
L3/4	146.2(32.5)	0.93(0.85–0.98)	8.4(4.2–12.7)	23.3(11.6–35.2)	15.9	4.5(3.9–5.3)
L4/5	136.5(41.3)	0.95(0.89–0.99)	9.1(4.5–13.9)	25.2(12.4–38.5)	18.4	6.6(5.7–7.8)
L5/S1	120.1(36.4)	0.88(0.74–0.97)	12.6(6.4–18.5)	35.0(17.7–51.3)	29.1	10.1(8.8–11.9)

Data are from the whole intervertebral disc (top panel: all slices averaged, entire disc) and nucleus (bottom panel: single central slice, 3^rd^ subregion). Mean, SEM, MD values are in ms. Global mean: Average value from all testing days, ICC: Intra-class correlation co-efficient, SEM: Standard error of the measurement, MD: Minimum detectable difference (also called minimum detectable change and indicates how large a difference needs to be present in a single participant for it to be detected as a “real effect” [23]), MD%: Minimum detectable difference expressed as a percentage of the mean value, CV: Co-efficient of variation; 95% CI: 95% confidence interval, AvLx: Average of all lumbar levels. For results of Bland-Altman testing, see S1 Table.

**Table 2:** Intra-discal subregion T_2_ and their between-day reliability.

Level	Global mean (SD)	ICC (95% CI)	SEM (95% CI)	MD (95% CI)	MD%	CV (95% CI)
*Anterior annulus (subregion 1)*						
AvLx	93.3(7.4)	0.74(0.52–0.93)	3.7(2.0–5.1)	10.4(5.5–14.2)	11.1	4.1(3.6–4.9)
L1/2	100.1(9.8)	0.60(0.34–0.87)	6.2(3.6–7.9)	17.3(9.9–22.0)	17.3	7.8(6.8–9.3)
L2/3	97.2(8.7)	0.64(0.40–0.89)	5.2(2.9–6.8)	14.4(8.0–18.8)	14.9	6.7(5.8–7.9)
L3/4	92.5(5.8)	0.37(0.14–0.74)	4.6(2.9–5.3)	12.7(8.1–14.8)	13.7	6.3(5.5–7.4)
L4/5	89.2(9.3)	0.35(0.13–0.72)	7.5(4.9–8.7)	20.8(13.5–24.0)	23.3	6.5(5.6–7.6)
L5/S1	87.3(10.5)	0.80(0.61–0.95)	4.6(2.4–6.5)	12.9(6.7–18.1)	14.7	5.7(4.9–6.7)
*Subregion 2*						
AvLx	99.7(15.3)	0.96(0.90–0.99)	3.2(1.6–5.0)	8.9(4.4–13.7)	9.0	2.6(2.3–3.1)
L1/2	102.0(16.2)	0.93(0.85–0.98)	4.2(2.1–6.3)	11.6(5.7–17.5)	11.3	4.5(3.9–5.3)
L2/3	101.9(14.4)	0.94(0.85–0.98)	3.6(1.8–5.5)	10.1(5.0–15.2)	9.9	3.6(3.2–4.3)
L3/4	102.3(17.2)	0.92(0.82–0.98)	4.9(2.4–7.3)	13.4(6.7–20.2)	13.1	3.5(3.1–4.2)
L4/5	94.6(21.8)	0.90(0.78–0.97)	6.9(3.5–10.3)	19.2(9.7–28.5)	20.3	3.5(3.0–4.1)
L5/S1	98.0(22.0)	0.97(0.93–0.99)	3.8(1.9–5.9)	10.6(5.2–16.4)	10.8	3.9(3.4–4.7)
*Central disc (subregion 3)*						
AvLx	114.1(22.0)	0.98(0.95–0.99)	3.2(1.6–5.1)	9.0(4.4–14.0)	7.9	2.6(2.2–3.0)
L1/2	115.0(23.9)	0.97(0.94–0.99)	3.9(1.9–6.1)	10.9(5.3–16.9)	9.4	3.4(2.9–4.0)
L2/3	121.8(24.9)	0.98(0.95–0.99)	3.7(1.8–5.7)	10.2(5.0–15.9)	8.4	3.1(2.7–3.7)
L3/4	119.4(23.7)	0.97(0.94–0.99)	3.8(1.8–5.8)	10.4(5.1–16.2)	8.7	2.9(2.5–3.4)
L4/5	110.5(27.9)	0.97(0.94–0.99)	4.5(2.2–7.1)	12.6(6.2–19.5)	11.4	3.4(3.0–4.0)
L5/S1	103.8(27.5)	0.99(0.97–1.00)	3.2(1.6–5.0)	8.9(4.4–14.0)	8.6	3.6(3.2–4.3)
*Subregion 4*						
AvLx	106.4(15.5)	0.97(0.93–0.99)	2.6(1.3–4.1)	7.3(3.6–11.3)	6.9	3.1(2.7–3.7)
L1/2	112.0(19.3)	0.97(0.93–0.99)	3.4(1.7–5.2)	9.4(4.6–14.5)	8.4	4.4(3.8–5.2)
L2/3	116.1(20.8)	0.97(0.92–0.99)	3.9(1.9–6.0)	10.7(5.3–16.5)	9.2	3.9(3.4–4.6)
L3/4	111.4(18.2)	0.92(0.82–0.98)	5.1(2.6–7.7)	14.2(7.1–21.3)	12.7	5.4(4.7–6.4)
L4/5	100.0(16.7)	0.95(0.89–0.99)	3.7(1.8–5.6)	10.2(5.0–15.7)	10.2	4.7(4.1–5.6)
L5/S1	92.4(19.8)	0.95(0.88–0.99)	4.5(2.2–6.9)	12.5(6.2–19.1)	13.6	5.6(4.9–6.6)
*Posterior annulus (subregion 5)*						
AvLx	80.9(5.0)	0.79(0.59–0.94)	2.3(1.2–3.2)	6.4(3.3–8.9)	7.9	3.4(3.0–4.1)
L1/2	83.7(8.3)	0.76(0.54–0.93)	4.1(2.2–5.6)	11.3(6.0–15.5)	13.5	6.0(5.2–7.1)
L2/3	81.4(5.9)	0.53(0.28–0.84)	4.0(2.4–5.0)	11.2(6.6–13.9)	13.8	7.6(6.6–9.0)
L3/4	80.7(5.5)	0.39(0.16–0.76)	4.3(2.7–5.0)	11.8(7.5–13.9)	14.6	8.6(7.5–10.1)
L4/5	78.3(5.2)	0.71(0.48–0.92)	2.8(1.5–3.7)	7.7(4.2–10.3)	9.8	4.7(4.0–5.5)
L5/S1	80.5(6.0)	0.61(0.36–0.87)	3.7(2.1–4.8)	10.3(5.9–13.2)	12.8	5.5(4.8–6.5)

Data are from subregions averaged across all slices. Mean, SEM, MD values are in ms. Global mean: Average value from all testing days, ICC: Intra-class correlation co-efficient, SEM: Standard error of the measurement, MD: Minimum detectable difference (also called minimum detectable change and indicates how large a difference needs to be present in a single participant for it to be detected as a “real effect” [23]), MD%: Minimum detectable difference expressed as a percentage of the mean value, CV: Co-efficient of variation; 95% CI: 95% confidence interval, AvLx: Average of all lumbar levels.

The correlation of T_2_ between different scanning time-points is presented in Table 3. Shorter duration correlations, such as between baseline and 34d post-baseline were typically higher (0.97 for the nucleus and 0.99 for the whole IVD) than between baseline and the final testing date 368d post-baseline (0.93 for the nucleus and 0.98 for the whole IVD).

**Table 3:** Correlation between T_2_ at each time-point for the whole intervertebral disc and the nucleus.

Testing time-point									
	Baseline	34d	60d	154d	188d	214d	308d	342d	368d
Baseline		0.99	0.98	0.99	0.99	0.99	0.99	0.99	0.98
34d	0.97		0.98	0.98	0.99	0.98	0.99	0.99	0.98
60d	0.94	0.96		0.99	0.98	0.99	0.97	0.98	0.97
154d	0.92	0.96	0.97		0.98	0.99	0.99	0.98	0.96
188d	0.95	0.97	0.98	0.95		0.99	0.99	0.98	0.98
214d	0.96	0.97	0.97	0.91	0.98		0.99	0.99	0.98
308d	0.95	0.96	0.92	0.91	0.94	0.96		0.99	0.98
342d	0.94	0.97	0.95	0.96	0.94	0.96	0.98		0.98
368d	0.93	0.94	0.94	0.96	0.95	0.96	0.95	0.96	

Data are Pearson’s correlation co-efficient from the whole intervertebral disc (top triangle in grey: all slices averaged, entire disc) and nucleus (bottom triangle: single central slice, 3^rd^ subregion).

### Comparison to Pfirrmann grade

**Fig 4:**
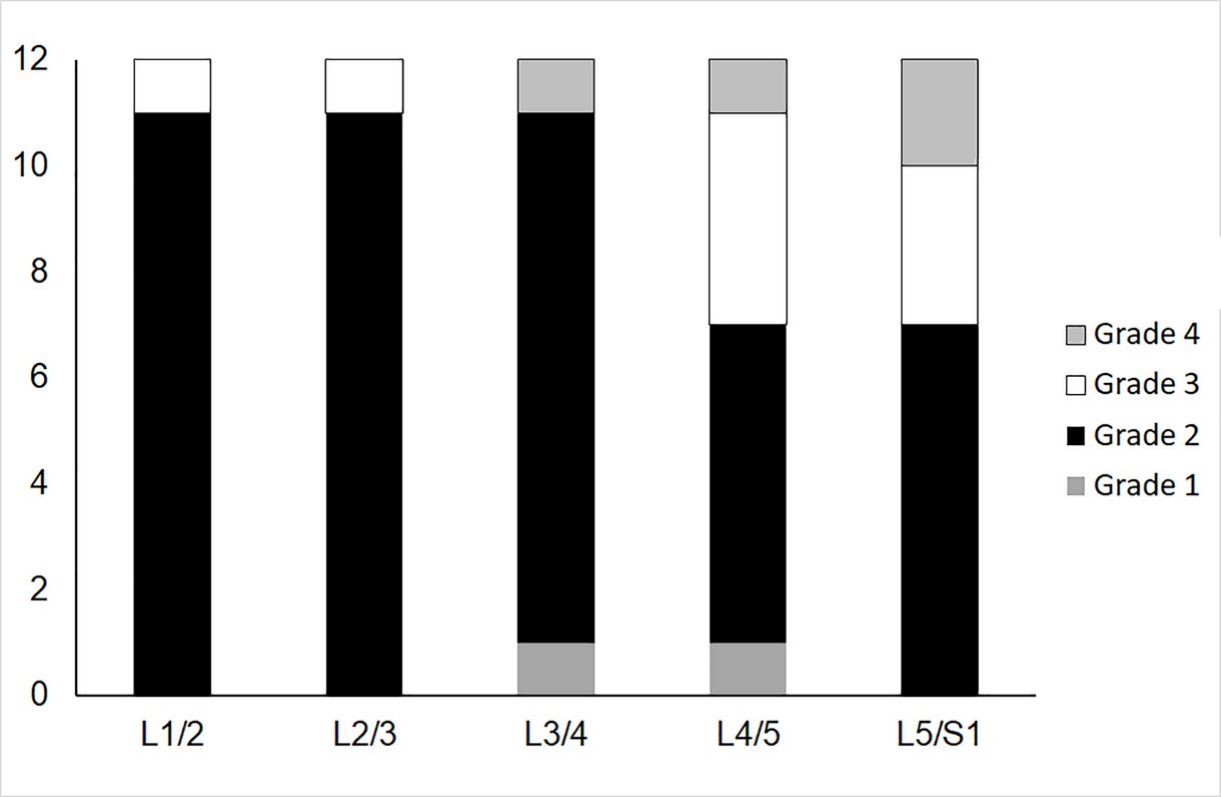
Number of intervertebral discs in each Pfirrmann Grade at each vertebral level. Due to the limited numbers of Grade 1 and Grade 4 intervertebral discs, analysis of reliability in different grades focuses on Grades 2 and 3 (see Results and Table 4).

There were, respectively, 2, 45, 9 and 4 grade 1, 2, 3 and 4 IVDs (Fig 4). The mean (SD) T2 of grade 2 IVDs was 107(11) ms and 82(5) ms of grade 3 IVDs. The ICCs and CVs of IVD T2 were lower in grade 3 IVDs as a whole as well as the nuclear region (Table 4).

**Table 4:** Repeatability depends on Pfirrmann grade.

Pfirrmann	Global mean (SD)	ICC (95% CI)	SEM (95% CI)	MD (95% CI)	MD%	CV (95% CI)
*Whole IVD*						
Grade 2	106.6(11.3)	0.96(0.94–0.98)	2.2(1.6–2.8)	6.1(4.5–7.8)	5.7	2.3(2.2–2.6)
Grade 3	81.8(5.3)	0.84(0.66–0.96)*	2.1(1.1–3.1)	6.0(3.1–8.5)	7.3	1.3(1.2–1.5)*
*Central slice*, *nuclear region*						
Grade 2	143.4(25.8)	0.91(0.85–0.95)	7.9(5.8–9.9)	21.8(16.1–27.4)	15.2	6.1(5.7–6.7)
Grade 3	95.4(15.9)	0.93(0.83–0.98)	4.3(2.2–6.5)*	12.0(6.0–18.1)*	12.6	2.9(2.7–3.1)*

Data are from the whole intervertebral disc (top panel: all slices averaged, entire disc) and nucleus (bottom panel: single central slice, 3^rd^ subregion). Mean, SEM, MD values are in ms. Global mean: Average value from all testing days, ICC: Intra-class correlation co-efficient, SEM: Standard error of the measurement, MD: Minimum detectable difference (also called minimum detectable change and indicates how large a difference needs to be present in a single participant for it to be detected as a “real effect” [23]), MD%: Minimum detectable difference expressed as a percentage of the mean value, CV: Co-efficient of variation, 95% CI: 95% confidence interval.

*: Mean of grade 3 value is outside the 95% CI of the value from grade 2 IVDs.

## Discussion

This study was the first to consider the long-term repeatability of quantitative MRI of the lumbar IVDs. We found lumbar IVD T_2_ to be stable over one year, with long-term ICCs greater than 0.90 for almost all regions of interest. An exception was the L5/S1 nucleus with an ICC of 0.88. Long-term CVs were also acceptable, typically being <5%.

The current study expands the knowledge base on techniques for quantifying the IVD and facilitates long-term studies using T_2_ as a key outcome measure. To date, the only reliability data available for IVD T_2_ was on ten participants measured approximately 10 to 15 minutes apart [13]. They found, similar to our work, that reliability of T_2_ quantification to be lowest at L5/S1 as compared to the remainder of the lumbar spine. Other studies of IVD measurement reliability have been performed on T2-weighed images, such as for morphology and signal intensity related parameters [27] and IVD height [28]. In both these cases, however, reliability was assessed by measuring the same images. A major component of measurement error comes from testing the same person on different days (such as with repositioning error of the patient in the MRI and of the slices in sequence setup).

Quantifying IVD signal properties as they relate to IVD composition, rather than IVD morphology, is likely more sensitive to detecting subtle changes. For example, water content losses occur progressively with age [29], whereas losses of disc height are less pronounced [30]. In the Pfirrmann [1] grading scheme, losses of height become pronounced in severe degeneration only, whereas stepwise reductions of signal intensity can be seen with each Pfirrmann grade [31], and IVD height is unlikely [32] an appropriate early indicator of IVD degeneration.

The findings of this study can have application immediately in research and potentially in the future in clinical practice. The findings here can be used in research when the T2 measurements are used at outcome measures: for sample size estimation in prospective studies and/or retrospective sensitivity analysis for existing data. Ultimately, in clinical practice, similar to current practice in the interpretation of bone mineral density changes over time [33], once developments in automated image analysis have progressed, the test-retest repeatability data here can inform whether a patient’s results over time exceed measurement error and thus potentially inform clinical management of that patients.

The strengths of the current study include its prospective design over a long period of time (12 months) and it is unique amongst method development studies for quantifying the lumbar IVDs, such as via T_2_. The main limitation of the current study is the loss to follow-up of participants beyond 154d post-baseline, which indicates that estimates of reliability beyond this time-point may be less robust. Another limitation relates to the use of a convenience sample of male participants in a wider project. Whilst we speculate reliability would be similar in clinical populations, such as in people with low back pain, we cannot be certain of this. Similarly, we have not examined individuals older than 45 years of age and did not examine women.

## Conclusions

In conclusion, T_2_ MRI was shown to produce reliable estimates of whole and regional lumbar IVD structure. This method was more reliable when averaging between vertebral levels, yet less reliable when IVDs had greater degeneration. These findings establish long-term repeatability of this method for future prospective studies with regards to design (i.e. sample size calculations) and implementation (i.e. quantification of changes over time).

## Supporting information

S1 Table Results from Bland-Altman testing. *: 0.008<p<0.026 and indicates raw p-value on regression testing for linear bias. None of these P-values remain significant after adjustment for multiple comparisons via the false discovery rate method. Note that all lower limits remain below zero and all upper limits above zero, indicating no systematic mean bias.
(PDF)
