# Supplementary material for: Quantitative assessment of the lumbar intervertebral disc via T2 shows excellent long-term reliability
Source: PLoS One. 2021 Apr 14;16(4):e0249855. doi: 10.1371/journal.pone.0249855 (PMC8046347; doi:10.1371/journal.pone.0249855)
Supplement: S1 Table — *: 0.008<p<0.026 and indicates raw p-value on regression testing for linear bias. None of these P-values remain significant after adjustment for multiple comparisons via the false discovery rate method. Note that all lower limits remain below zero and all upper limits above zero, indicating no systematic mean bias. (PDF) [file pone.0249855.s001.pdf]

S1 Table. Results from Bland-Altman testing.

**Level**	**Mean difference(limits of agreement) versus baseline**							
	34d	56d	176d	210d	266d	386d	420d	476d
Whole IVD								
AvLx	1.3(-8,11)	0.0(-14,13)	-1.0(-15,13)	0.5(-16,17)*	-0.9(-18,16)*	0.7(-11,13)	-1.9(-14,10)	6.8(-5,19)
L1/2	-0.6(-10,9)	-3.1(-13,7)	-3.3(-12,5)	-2.6(-11,6)	-2.5(-11,7)	-1.5(-12,9)	-1.5(-11,8)	0.7(-8,9)
L2/3	-1.5(-12,9)	0.1(-9,9)	-1.7(-16,13)	1.5(-13,17)	1.9(-7,11)	0.8(-8,10)	1.5(-8,11)	-1.1(-12,10)
L3/4	-1.6(-14,11)	0.1(-13,13)	-5.4(-24,13)	3.2(-13,20)	2.5(-14,18)	-1.1(-16,14)	-1.9(-8,4)	-2.7(-16,10)
L4/5	-0.1(-12,12)	0.9(-10,12)	-3.1(-19,13)	3.1(-14,20)	3.4(-10,17)	0.6(-18,19)	3.3(-9,15)	0.7(-20,21)
L5/S1	-3.2(-20,13)	-3.2(-13,7)	-3.3(-16,10)	-2.4(-19,14)	-0.3(-15,15)	-0.7(-19,18)	-1.1(-20,18)	-2.0(-20,15)
Central slice, nuclear region								
AvLx	-0.3(-25,24)	5.5(-25,37)	-7.6(-45,30)*	-2.3(-54,50)*	-3.3(-47,40)	-1.0(-42,40)	-1.9(-48,44)	-1.1(-4,2)
L1/2	1.5(-26,29)	-0.9(-48,46)	-3.2(-35,28)	-5.9(-62,50)	-9.9(-48,28)	-0.5(-33,32)	3.3(-37,43)	1.2(-32,34)
L2/3	-1.9(-21,17)	-2.7(-16,11)	1.1(-26,28)	-3.0(-15,9)	-6.3(-31,19)	-2.5(-16,11)*	1.5(-11,14)	1.6(-9,12)
L3/4	0.0(-17,17)	1.0(-32,34)	0.2(-38,38)	0.4(-28,29)	-1.7(-35,31)	1.5(-21,24)	4.6(-29,39)	4.2(-23,31)
L4/5	1.7(-21,25)	-3.0(-21,15)	-0.4(-23,22)	-2.3(-16,12)	-1.6(-30,26)	2.0(-15,19)	5.9(-8,20)	3.6(-9,16)
L5/S1	-4.1(-33,25)	-5.0(-20,10)	0.5(-33,34)	-2.3(-25,20)*	-8.5(-45,28)	-0.1(-19,18)	3.3(-9,16)*	2.9(-7,13)

*: $0.008<p<0.026$ and indicates raw p-value on regression testing for linear bias. None of these P-values remain significant after adjustment for multiple comparisons via the false discovery rate method. Note that all lower limits remain below zero and all upper limits above zero, indicating no systematic mean bias.
